# Next-Gen ^18^F-Relay Reagent: Optimising [^18^F]Ethanesulfonyl Fluoride

**DOI:** 10.3390/ijms27093982

**Published:** 2026-04-29

**Authors:** Margaret L. Aulsebrook, Giancarlo Pascali, Kellie L. Tuck, Parisa T. Rashid, Manja Kubeil, Christoph E. Hagemeyer, Jens Pietzsch, Markus Laube

**Affiliations:** 1 Monash Biomedical Imaging, Monash University, Melbourne, VIC 3800, Australia; 2 Australian Nuclear Science and Technology Organisation (ANSTO), Sydney, NSW 2234, Australia; 3 School of Chemistry, Monash University, Melbourne, VIC 3800, Australia; 4 Helmholtz-Zentrum Dresden-Rossendorf, Institute of Radiopharmaceutical Cancer Research, 01328 Dresden, Germany; 5 Helmholtz-Zentrum Dresden-Rossendorf, Institute of Resource Ecology, 01328 Dresden, Germany; 6 School of Translational Medicine, Monash University, Melbourne, VIC 3004, Australia; 7 Faculty of Chemistry and Food Chemistry, School of Science, Technische Universität Dresden, 01062 Dresden, Germany

**Keywords:** fluorine, radiofluorination, positron emission tomography, radiopharmaceuticals, sulfonyl fluoride

## Abstract

Conventional nucleophilic radiofluorination requires azeotropic drying to generate reactive [^18^F]fluoride, introducing time delays and activity losses. [^18^F]Fluoride relay reagents such as [^18^F]triflyl fluoride (**[^18^F]TfF**) and [^18^F]ethenesulfonyl fluoride (**[^18^F]E=SF**) have recently emerged as efficient alternatives that bypass this step. Here, we introduce [^18^F]ethanesulfonyl fluoride (**[^18^F]E-SF**) as a new relay reagent and benchmark its production and radiolabelling performance against **[^18^F]TfF** and **[^18^F]E=SF**. **[^18^F]E-SF** was prepared from commercially available 2,4,6-trichlorophenyl-1-ethanesulfonate (TCP-ethane) using a microlitre-scale radiofluorination approach, enabling direct distillation and SPE trapping of the product. Under optimised conditions, **[^18^F]E-SF** was obtained in 76 ± 23% RCY (*n* = 6), compared with 27 ± 6% (*n* = 2) for **[^18^F]E=SF** and up to 97 ± 2% (*n* = 3) for **[^18^F]TfF** using an optimised literature-based protocol. Subsequent model labelling reactions demonstrated effective aliphatic nucleophilic substitution to [^18^F]fluoroethyl tosylate ([^18^F]FEtOTs) and aromatic nucleophilic substitution to [^18^F]fluorobenzaldehyde with high radiochemical conversion. These results establish **[^18^F]E-SF** as a robust and operationally simple relay reagent with high production yields from a commercially available precursor. It is compatible with SPE trapping and achieves production yields comparable to **[^18^F]E=SF** and **[^18^F]TfF**, respectively, warranting future automated production for supporting the potential use of **[^18^F]E-SF** in streamlined and decentralised ^18^F-labelling workflows.

## 1. Introduction

Fluorine-18 is a vital radionuclide for positron emission tomography (PET), a highly sensitive and quantitative molecular imaging modality. The favourable physical properties of fluorine-18, including an optimal half-life, high isotopic purity, low positron energy, and reliable cyclotron production, have made it integral to the majority of clinical PET scans conducted daily around the globe [1,2]. Further, radiochemistry with ^18^F is widely explored [3,4] and several labelling methods exist, like common nucleophilic (aromatic) substitution or copper-mediated radiofluorination for carbon–fluorine bond formation, or alternative ^18^F-labelling strategies based on F–heteroatom bond formation (e.g., B–F, Si–F, S–F, Al–F). Despite these advantages, experimental challenges exist, and access to fluorine-18 and, consequently, to fluorine-18-labelled radiopharmaceuticals remains constrained. On the one hand, production is largely centralised in a small number of cyclotron facilities, reflecting the high capital cost, specialised infrastructure, and operational expertise required to access these reagents [5]. Clinical sites are therefore dependent on the schedule, product range, and distribution capacity of their supplying cyclotron and radiopharmaceutical manufacturing site, limiting flexibility and restricting availability, particularly in rural and remote regions, e.g., of the U.S. or Australia, where inequities in healthcare access are already well documented [6,7,8]. [^18^F]Fluoride produced on a cyclotron is delivered in an oxygen-18-enriched water matrix, which is unsuitable for direct labelling. While solid-phase extraction (SPE) using anion-exchange cartridges and azeotropic drying of [^18^F]fluoride for water removal are standardised procedures today to obtain reactive [^18^F]fluoride, this step introduces commonly strongly basic conditions incompatible with base-sensitive substrates, time delays and potentially variances in residual water content and, hence, radiochemical yield.

**Figure 1:**
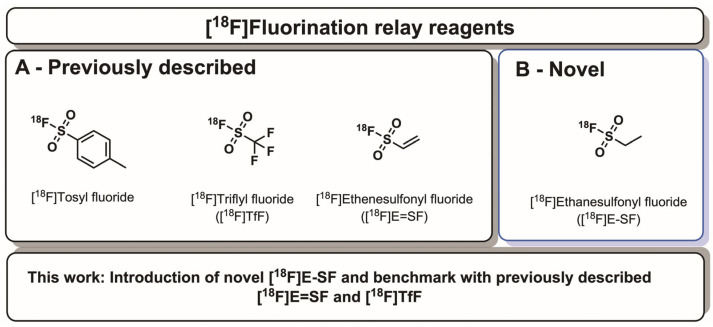
Overview and aim of this work: (**A**) Previously reported radiofluorination relay reagents: [^18^F]tosyl fluoride, [^18^F]triflyl fluoride, and [^18^F]ethenesulfonyl fluoride. (**B**) [^18^F]ethanesulfonyl fluoride as a novel radiofluorination relay reagent reported in this work.

The challenges of [^18^F]fluoride chemistry have stimulated interest in the development of new elution protocols [9,10,11], radiofluorination techniques on heteroatoms like [^18^F]AlF, [^18^F]SiFA [3,4] or [^18^F]SF_5_ [12], and ^18^F-relay reagents as an emerging class of intermediates, which address a distinct and critical bottleneck in this process: the generation, handling, and deployment of reactive [^18^F]fluoride. In the relay agent approach, cyclotron-produced [^18^F]fluoride is easily converted into an ^18^F-relay reagent, which is a stable, isolable, and even transportable intermediate that can liberate [^18^F]fluoride for use in radiolabelling. When such a product can be isolated and transported from a central production site, it equips laboratories with more modest infrastructure to manufacture radiofluorinated tracers [13]. The power of these reagents is beginning to be tested with an increasing arsenal reported within the literature, including [^18^F]tosyl fluoride [14], [^18^F]triflyl fluoride (**[^18^F]TfF**) [15,16], and [^18^F]ethenesulfonyl fluoride (**[^18^F]E=SF**) [13,17] as prominent examples (Figure 1A). [^18^F]Tosyl fluoride [14] is generated on a cartridge from [^18^F]fluoride and tosyl chloride using a peristaltic pump setup and can be stored after elution as a solution for later liberation of [^18^F]fluoride. [^18^F]Triflyl fluoride (**[^18^F]TfF**) [15,16] is synthesised from [^18^F]fluoride and *N*-phenyl-bis(trifluoromethanesulfonimide) in aqueous media and transported as a gaseous relay reagent to a second reaction vial for the liberation of [^18^F]fluoride, circumventing the need for azeotropic drying. [^18^F]Ethenesulfonyl fluoride (**[^18^F]E=SF**) [13,17] is easily synthesised from [^18^F]fluoride and 2,4,6-trichlorophenyl ethene sulfonate, and, additionally, allows for its trapping on a Silica SPE cartridge for subsequent transport and final liberation of [^18^F]fluoride at a different site, supporting decentralised and simplified labelling workflows. For all three reagents, [^18^F]fluoride can be released under controlled conditions with minimal base amounts, and in the case of **[^18^F]E=SF**, just by eluting the Si cartridge with any solvent, rendering them potentially useful for base-sensitive precursors and simplifying time-critical fluoride processing before labelling, lowering the operational and technical barriers associated with handling ^18^F. Thereby, these reagents broaden access to PET radiochemistry and enable more flexible, decentralised tracer production.

As part of our efforts to produce novel radiotracers, we were interested in establishing the reported relay reagents [^18^F]triflyl fluoride (**[^18^F]TfF**) [15,16] and [^18^F]ethenesulfonyl fluoride (**[^18^F]E=SF**) in-house, identifying [^18^F]ethanesulfonyl fluoride (**[^18^F]E-SF**, Figure 1B) as an attractive alternative and novel ^18^F-relay reagent based on the availability of a commercial precursor, and evaluating its radiosynthesis and reactivity. Herein, we report insights from these efforts and optimisation results, as well as detail **[^18^F]E-SF** as a useful next-generation ^18^F-relay reagent.

## 2. Results

### 2.1. [^18^F]Triflyl Fluoride ([^18^F[TfF)

**Figure 2:**
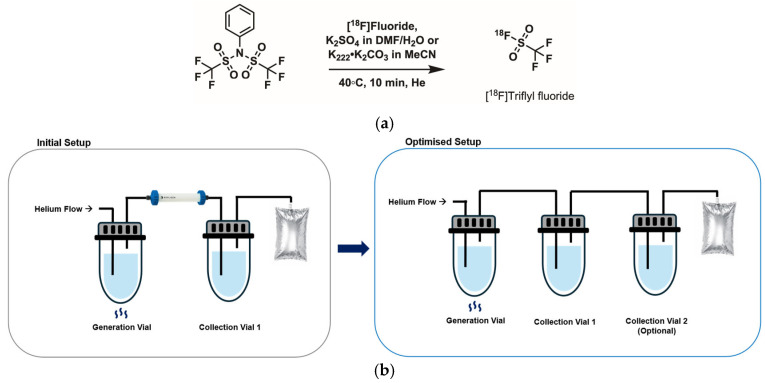
(**a**) Radiosynthesis; (**b**) initial and optimised production scheme for the generation of **[^18^F]TfF**.

We started our attempts towards ^18^F-relay reagents following the literature procedure for the generation of **[^18^F]TfF**, which involved elution of [^18^F]fluoride using a 0.1 M potassium sulfate solution and dimethylformamide (DMF), followed by the generation of **[^18^F]TfF** through the addition of *N*-phenyl-bis(trifluoromethanesulfonimide) as precursor and reaction at 40 °C (Figure 2a). The **[^18^F]TfF** product was then carried out of the reaction vessel (Figure 2b, left) with a helium stream (10 mL/min) over 10 min and passed through a phosphorus pentoxide-filled drying column before being trapped in a downstream collection vial containing Kryptofix 2.2.2 (K_222_) and potassium bicarbonate (KHCO_3_) in acetonitrile (MeCN) [16]. In our hands, this procedure gave inconsistent results with radiochemical yields (RCYs) of 26% and 2%, respectively, which may reflect the sensitivity of the method to subtle differences in experimental conditions between laboratories, such as reactor geometry, gas flow, or reagent preparation. This prompted us to test our hydrous ^18^F-fluorination approach initially described for fluoroethylation [18], which allows radiosynthesis, e.g., of [^18^F]fluoroethyl tosylate, without azeotropic drying. Herein, we eluted the [^18^F]fluoride from the QMA with a solution suitable for radiolabelling, either HYFE (i.e., 58.9 mM K_222_/29.4 mM K_2_CO_3_ dissolved in only 2% water in MeCN) or HYFE ¼ (i.e., 7.3 mM K_2_CO_3_ in 3% water in MeCN). Because only a minimal amount of water was present and the reaction was performed at 40 °C only, we envisaged removing the phosphorus pentoxide column to further ease the system (Figure 2b, right). To our delight, using both HYFE and HYFE ¼ without any drying, we observed high RCYs of 72 ± 22% (*n* = 3) and 97 ± 2% (*n* = 3), respectively, in 5–10 min (Table 1). Of note, two collection vials containing K_222_^.^KHCO_3_ in MeCN were initially installed as a safety measure to capture any radioactivity escaping the first vial, but **[^18^F]TfF** was quantitatively trapped in the first vial, so the terminal vial proved unnecessary.

**Figure 3:**
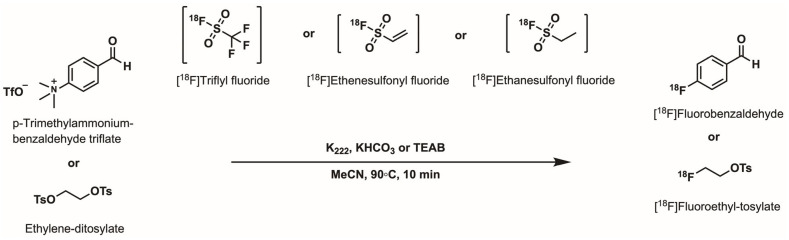
Radiosynthesis of [^18^F]fluorobenzaldehyde or [^18^F]FEtOTs using **[^18^F]TfF**, **[^18^F]E=SF** or **[^18^F]E-SF** and K_222_.KHCO_3_ or TEAB, as indicated in Table 2, Table 4 and Table 6.

In order to establish the reactivity profile of the obtained relay reagent, we aimed to perform test labelling with **[^18^F]TfF**. Given the widespread use of both aliphatic and aromatic nucleophilic substitution pathways in radiofluorination, ethylene ditosylate (EtdiTos) forming [^18^F]fluoroethyl tosylate ([^18^F]FEtOTs) and *p*-trimethylammonium benzaldehyde triflate (TMBATf) forming [^18^F]fluorobenzaldehyde (Figure 3) were selected as representative substrates and also for testing the other ^18^F-relay reagents described hereafter. Both reactions were performed at 90 °C for 10 min using the microlitre scale radiofluorination approach in HPLC vials [19] and proceeded with high efficiency, affording [^18^F]FEtOTs in 76 ± 1% radiochemical conversion (RCC) and [^18^F]fluorobenzaldehyde in RCC values of 41 ± 4% (obtained starting from HYFE) and 50 ± 4% (obtained starting from HYFE ¼) (Table 2). These results reflect the established reactivity trends in nucleophilic ^18^F chemistry and provide a benchmark for comparing the performance of alternative ^18^F-relay reagents. For context, these results also compare favourably to the initial literature reports, which noted an RCC for the aliphatic nucleophilic substitution of 57% towards [^18^F]FES and only 10% towards [^18^F]FET [16].

In conclusion, the optimised procedure for **[^18^F]TfF** production compares well to the method reported by Pees et al. [16]. It is operationally straightforward, uses standard reagents and a QMA cartridge to align closely with conventional ^18^F radiochemistry, and provides the relay reagent in high yield and reactivity.

### 2.2. [^18^F]Ethenesulfonyl Fluoride ([^18^F]E=SF)

**Figure 4:**
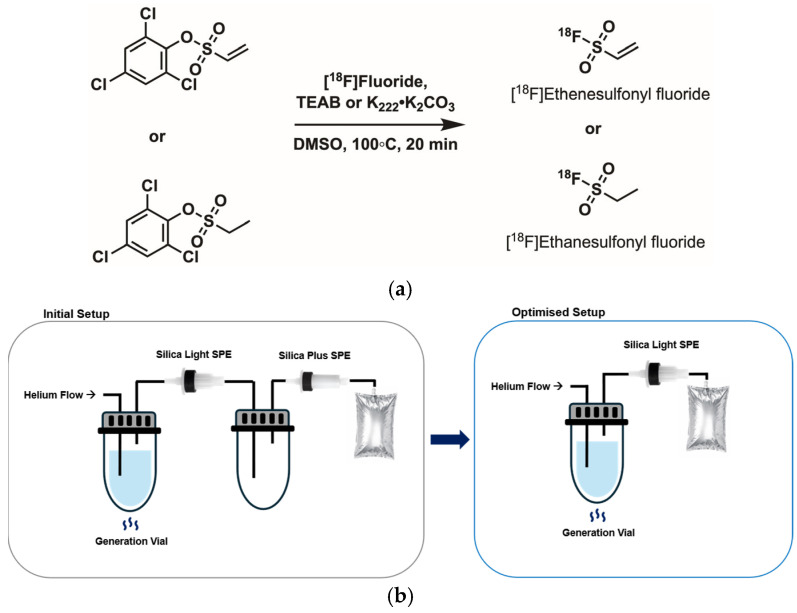
(**a**) Radiosynthesis; (**b**) initial and optimised production scheme for the generation of **[^18^F]E=SF** and **[^18^F]E-SF**.

Next, we attempted the synthesis of **[^18^F]E=SF** (Figure 4a), which allows for transferring and trapping on Silica Plus SPE cartridges [17]. 2,4,6-Trichlorophenyl ethenesulfonate (TCP-ethene) was synthesised according to Liu et al. (2023) [20] and Zeng et al. (2022) [21] in 48% yield as a precursor for the relay reagent. The original paper used non-dried [^18^F]TEAF in a microfluidic reactor environment; given that we used a microlitre-scale radiofluorination approach in HPLC vials [19], we opted for a more conventional reaction of azeotropically dried tetraethylammonium [^18^F]fluoride with TCP-ethene in dimethyl sulfoxide (DMSO) (20 mg/mL) at 100 °C. **[^18^F]E=SF** was carried out of the reactor under N_2_ flow (0.15 L/min) over 20 min through an SEP Pak^®^ Silica Plus light cartridge, into an empty reactor and towards a terminal SEP Pak^®^ Silica Plus Long cartridge (Figure 4b, left). The previous literature reported quantitative trapping of **[^18^F]E=SF** on a Silica Plus Long cartridge; in contrast, we observed the majority of trapped **[^18^F]E=SF** on the first, with an RCY of 28%, but not on the second (RCY 5%) Silica Plus SPE cartridge (Table 3). Further, the RCY was considerably lower in these initial attempts than the 57 ± 11% obtained using the original microfluidic setting [17].

These results again prompted us to test K_222_^.^K_2_CO_3_ applied as an HYFE solution [18] for this reaction, as it allows for labelling in moisture-sensitive reactions after one solvent dry-down, without needing consecutive azeotropic drying steps. Applying QMA elution with HYFE, single dry-down and analogous reaction conditions afforded **[^18^F]E=SF** at a comparable RCY of 27 ± 6% (*n* = 2, Table 3). Of note, because we observed trapping mainly in the first Silica Short SPE, we optimised the setting so that the SPE was connected to the reaction vial by a needle from the male side and vented into a gas bag as a safety measure (Figure 4b, right). After completing the **[^18^F]E=SF** transfer, this allowed us to quickly take out the SPE with a needle, disconnect the line to the gas bag on the female side and elute the SPE in reverse from the female side, which improved handling and elution efficacy. As a further side note, no conversion was obtained when the precursor concentration was lowered to a final concentration of 2 mg/mL instead of 20 mg/mL.

To test the reactivity of **[^18^F]E=SF**, EtdiTos was chosen as a model substrate and reacted at 90 °C in MeCN using either tetraethylammonium bicarbonate (TEAB) or KHCO_3_^.^K_222_ as bases (Table 4). This proved the high reactivity of the relay reagent, with an RCC of 63 ± 10% (*n* = 6), showing a slightly higher RCC for the KHCO_3_^.^K_222_ subset. For comparison, the reported reactivity of **[^18^F]E=SF** [17] is given with an RCC of 76% for the [^18^F]FDG precursor and 85% and 75% for structurally complex aliphatic -OTs substrates, highlighting that the obtained **[^18^F]E=SF** relay reagent achieved comparable labelling efficiency.

While lower RCY values were obtained for **[^18^F]E=SF** compared to the reported microfluidic radiosynthesis, the relay reagent could be synthesised with acceptable yield and high reactivity in the manual radiosynthesis attempts reported herein.

### 2.3. [^18^F]Ethanesulfonyl Fluoride ([^18^F]E-SF)

Owing to its structural similarity to TCP-ethene and commercial availability, 2,4,6-trichlorophenyl ethanesulfonate (TCP-ethane) was investigated to synthesise **[^18^F]E-SF** as a potential ^18^F-relay reagent (Figure 4a). Both the initial and optimised experimental setup used for **[^18^F]E=SF** production were applied to **[^18^F]E-SF** (Figure 4b), starting either from azeotropically dried tetraethylammonium [^18^F]fluoride or a [^18^F]KF^.^K_222_ complex after single-solvent dry-down with an HYFE solution. For both, TCP-ethane (20 mg/mL) in DMSO was then reacted with [^18^F]fluoride at 110 °C under helium flow for 20 min. To our delight, **[^18^F]E-SF** was efficiently trapped on the first Silica Plus light cartridge (Table 5) with a high RCY of 68 ± 12% (*n* = 2) when synthesised from tetraethylammonium [^18^F]fluoride and with 76 ± 23% (*n* = 6) when synthesised using the HYFE solution (for detailed RCY results, see Supplementary Information Table S1). Of note, the high standard deviation for the latter was a result of one experiment with a considerably lower RCY of 28% without a clarified reason, so we decided to include this value. Elution of **[^18^F]E-SF** from both cartridges with MeCN was consistently high, with elution efficiencies exceeding 96%. Subsequent labelling with EtdiTos and TMBATf (Figure 3) was performed as described above. [^18^F]FEtOTs was obtained at 39–43% and 58–72% RCC using TEAB and KHCO_3_^.^K_222_, respectively, while [^18^F]fluorobenzaldehyde was obtained from both preparation methods at 60 ± 3% and 72 ± 12% RCC using TEAB as the base (Table 6), which confirmed the high reactivity of the novel ^18^F-relay reagent. Overall, these findings demonstrate a higher production efficiency and comparable reactivity of **[^18^F]E-SF** compared with **[^18^F]E=SF** in the applied setup.

## 3. Discussion

When considering all three relay reagents, **[^18^F]E-SF** emerges as a particularly promising alternative. **[^18^F]TfF** can be obtained within short synthesis times (<10 min), but its preparation requires precise handling and careful distillation, and it appears to be sensitive to subtle variations in experimental conditions. Small differences in water content, gas-flow stability, or reactor configuration led to inconsistent outcomes in our experiments, suggesting that the chemistry may be vulnerable to lab-to-lab variability despite the high yields reported in the literature. Therefore, we developed an alternative approach with a straightforward preparation method for manual synthesis of **[^18^F]TfF** in high radiochemical yields (up to 97 ± 2% (*n* = 3)). **[^18^F]E=SF**, while operationally simpler and amenable to direct SPE trapping, suffered in our experiments from significantly lower overall yields (~28%) using both the initial and optimised setup, the latter marked by direct trapping of the ^18^F-relay reagent on the first SPE cartridge. However, its labelling capacity was verified and in line with the literature reports. In contrast, **[^18^F]E-SF** combines the operational simplicity of the optimised **[^18^F]E=SF** production with yields and trapping efficiencies (RCY 76 ± 23% (*n* = 6)) approaching those of **[^18^F]TfF** (RCY up to 97 ± 2% (*n* = 3)). In contrast with TfF (−25 °C), the reported boiling points of E=SF (119 °C [22]) and E-SF (110 °C [23]) allow their transfer and trapping on SPE cartridges; in addition, they demonstrated similar high SPE capture and elution efficiency (>95%), as well as reliable radiolabelling of both aliphatic and aromatic compounds under both KHCO_3_^.^K_222_ and TEAB activation. As an important practical consideration, a TCP-ethane precursor is commercially available, eliminating the synthetic burden associated with generating **[^18^F]E=SF,** also taking the need for a high TCP-ethene precursor concentration for labelling into account. Although all experiments were conducted by manual syntheses at this point, the optimised methods are marked by excellent handling characteristics, with consistent SPE retention and reactivity of the ^18^F-relay reagents, which warrants future automation and scale-up.

## 4. Materials and Methods

Unless otherwise stated, all reagents and solvents were obtained from commercial vendors and used without further purification. For radiolabelling, MeCN, DMF and DMSO (anhydrous over a molecular sieve; Sigma Aldrich, Taufkirchen, Germany) were used.

All NMR spectra were recorded at 25 °C using a Bruker Avance III 400 MHz/Agilent DD2-400 MHz (Bruker BioSpin GmbH & Co. KG, Ettlingen, Germany; ^1^H: 400 MHz, ^13^C: 101 MHz, ^19^F: 376 MHz). The evaluation of the NMR spectra was carried out using the program Mestrelab MestReNova (version 15.0.1).

No-carrier-added aqueous [^18^F]fluoride was produced in a TR-FLEX 18–30 MeV cyclotron (ACSI, Richmond/Vancouver, BC, Canada) by irradiation of [^18^O]H_2_O via an ^18^O(p,n)^18^F nuclear reaction. Analytical radio-HPLC was performed with the following system. Agilent 1100 HPLC (Agilent Technologies, Santa Clara, CA, USA): binary pump G1312A; auto sampler G1313A; column oven G1316A; degasser G1322A; UV detector G1314A; γ detector Gabi Star^®^ (Raytest Isotopenmeßgeräte GmbH, Straubenhardt, Germany); column X-Terra MS C18 (Waters, Milford, MA, USA; 2.5 µm 50 × 3 mm), with a column temperature of 40 °C; (A) MeCN/(B) 0.1% trifluoroacetic acid (TFA) in H_2_O; flow rate 0.6 mL/min; and gradient: *t*_0 min_ 5/95–*t*_0.3 min_ 5/95–*t*_5.3 min_ 95/5–*t*_7.0 min_ 95/5–*t*_8.0 min_ 5/95–*t*_12.0 min_ 5/95). The products were monitored at λ = 254 nm with the γ detector. The t_R_ value for [^18^F]fluorobenzaldehyde was 4.4 min. Radio-thin layer chromatography (radio-TLC) was performed on silica gel F-254 aluminium plates (Merck KGaA, Darmstadt, Germany; TLC silica gel 60 F_254_, 1.05554.0001) with *n*-hexane:ethyl acetate, 1:1 (*v*/*v*), as the eluent. Visualisation was carried out using a CR35bio scanner system (Raytest, Straubenhardt, Germany) and analysed using advanced image data analyser (AIDA, version 5.1 SP4) software. The R*_f_* value for [^18^F]fluoroethyl tosylate was 0.5. The radiochemical yields of the ^18^F-relay reagents were determined by comparing the trapped ([^18^F]TfF) or SPE eluted ^18^F activity with the starting activity prior to precursor addition. The radiochemical conversion was determined by radio-TLC or radio-HPLC as the ratio of the product peak area to the total area of radioactive peaks in the chromatogram.

Caution: Handling [^18^F]triflyl fluoride, [^18^F]ethenesulfonyl fluoride, and [^18^F]ethanesulfonyl fluoride involves the generation and transfer of radioactive gaseous intermediates and should only be performed with the respective precautions in a radionuclide hood or hot cell. Precautions include testing equipment and transfer lines for gas leak rates, which should be as low as possible (e.g., <2 mL/min at 1.8 bar line pressure), directed ventilation, radiation monitoring and installation of gas waste bags or liquid traps.

For preparation, a Sep-Pak light Accell Plus light QMA^®^ cartridge (46 mg, Waters, Milford, MA, USA) was washed with water (10 mL), 1 M KHCO_3_ (10 mL) and water (10 mL) using an ÄKTA prime device before use. Chromafix 30 PS-HCO_3_ SPE (Macherey Nagel, Düren, Germany) was washed with water (10 mL). SEP Pak^®^ Silica Plus Light SPE (120 mg, Waters, Milford, MA, USA) and SEP Pak^®^ Silica Plus Long SPE (690 mg, Waters, Milford, MA, USA) were used without further equilibration.

### 4.1. Radiosynthesis

#### 4.1.1. [^18^F]Triflyl Fluoride ([^18^F]TfF)

Manual radiosynthesis of [^18^F]Triflyl fluoride (**[^18^F]TfF**) according to Pees et al. [16]: A collection vial was equipped beforehand with 100 µL of a 266 mM stock of Kryptofix 222/potassium bicarbonate (K_222_^.^KHCO_3_) in MeCN and 800–900 µL MeCN. Cyclotron-produced [^18^F]fluoride was trapped on a Chromafix 30-PS-HCO_3_ cartridge and eluted into the ‘generation vial’ using a 0.1 M potassium sulfate solution (500 μL) followed by DMF (850 μL), with a confirmed high elution efficiency. To produce **[^18^F]TfF**, the resulting solution was treated directly with a 0.1 M *N*-phenyl-bis(trifluoromethanesulfonimide) precursor (150 μL); the vial was closed and connected to the transfer lines. The mixture was then heated at 40 °C for 13 min or 60–70 °C for 10 min. The **[^18^F]TfF** product was carried out of the reaction vessel using a phosphorus pentoxide column into the collection vial containing K_222_^.^KHCO_3_ in MeCN with a helium stream (10 mL/min) over the reaction time. The radiochemical yields (RCYs) for the production and transfer into the collection vial were found to be 26% and 2%, respectively.

Optimised synthesis of [^18^F]triflyl fluoride (**[^18^F]TfF**): A collection vial was equipped beforehand with 100 µL of a 266 mM stock of K_222_^.^KHCO_3_ in MeCN and 800–900 µL MeCN. Cyclotron-produced [^18^F]fluoride (20–200 MBq) was trapped on a QMA cartridge, washed with 1 mL MeCN and eluted with either 1 mL K_222_^.^K_2_CO_3_ solution containing 29.4 mM K_2_CO_3_ and 58.9 mM K_222_ in MeCN/water (98/2) (hydrous fluoroethylation mixture [18], HYFE solution) or 1 mL K_222_^.^K_2_CO_3_ solution containing 7.3 mM K_2_CO_3_ and 58.9 mM K_222_ in MeCN/water (97/3) (HYFE ¼ solution). An aliquot of the eluted [^18^F]fluoride (commonly 0.5 mL) was reacted without azeotropic drying directly with an equal volume of the *N*-phenyl-bis(trifluoromethanesulfonimide) solution (3.8 mg in 0.8 mL MeCN) at 40 °C for 5–10 min while applying a helium stream (10 mL/min) to carry **[^18^F]TfF** directly into the collection vial containing K_222_^.^KHCO_3_ in MeCN. **[^18^F]TfF** was obtained at an RCY of 97 ± 2% (*n* = 3) in 10 min using HYFE ¼ and an RCY of 72 ± 22% (*n* = 3) in 5–10 min using the HYFE solution (Table 1).

For labelling (each *n* = 3) using our recently described microlitre scale radiofluorination approach in HPLC vials (1.5 mL screw top vial, Agilent) [19], aliquots of the collected **[^18^F]TfF** (50 µL) were taken, mixed with 25 µL of either *p*-trimethylammonium-benzaldehyde triflate (TMBATf, 10 mg/mL in MeCN) or ethylene ditosylate (EtdiTos, 10 mg/mL in MeCN) and heated at 90 °C for 10 min, followed by deactivation and dilution with 100–500 µL MeCN/H_2_O and radio-TLC or radio-HPLC to analyse for radiochemical conversion (RCC, Table 2). Of note, for an experiment performed at a conventional scale (0.8 mL of ^18^F with 0.4 mL EtdiTos), quenched with 1.5 mL MeCN/H_2_O and analysed, a comparable RCC of 73% was observed.

#### 4.1.2. [^18^F]Ethenesulfonyl Fluoride ([^18^F]E=SF)

Manual radiosynthesis of [^18^F]ethenesulfonyl fluoride (**[^18^F]E=SF**) according to Zhang et al. [17]: Cyclotron-produced [^18^F]fluoride was trapped on a QMA cartridge and eluted into a ‘generation vial’ using a 0.075 M tetraethylammonium bicarbonate (TEAB) solution in 90% MeCN/10% H_2_O (800 μL) with a confirmed high elution efficiency. Azeotropic drying of [^18^F]fluoride containing eluate (100 µL) was performed at 110 °C, applying a stream of helium, including two consecutive additions of MeCN (100 µL). After completion of the drying step, the vial was connected to a Silica Plus Light cartridge, followed by an empty reactor, Silica Plus Long column and a final gas waste bag. Then, 2,4,6-trichlorophenyl ethenesulfonate (TCP-ethene, 100 µL, 20 mg/mL in DMSO) was added, the closed mixture was reacted at 100 °C, and the product was carried out of the reactor under N_2_ flow (0.15 L/min) over a time course of 20 min. In contrast to the reported procedure, the majority of **[^18^F]E=SF** was captured on the first (short, RCY 28%) but not the second (plus, RCY 5%) silica SPE cartridge. **[^18^F]E=SF** was eluted with MeCN (1 mL for short cartridge, 2 mL for long cartridge) from both cartridges with an elution efficiency of 83–85%.

Optimised radiosynthesis of [^18^F]ethenesulfonyl fluoride (**[^18^F]E=SF**): Cyclotron-produced [^18^F]fluoride (400–700 MBq) was trapped on a waters QMA SPE cartridge, washed with 1 mL MeCN and eluted with 1 mL HYFE solution. An aliquot of the eluted [^18^F]fluoride (100 µL) was dried down at 90 °C for 3–4 min, applying a stream of helium, and 2,4,6-trichlorophenyl ethenesulfonate (TCP-ethene, 100 µL, 20 mg/mL in DMSO) was added. The closed vial was connected to a Silica Light SPE cartridge connected towards the reactor from the male side by a needle, followed by a transfer line to the final gas waste bag. The mixture was reacted at 100 °C, and the product was carried out of the reactor under N_2_ flow (0.15 L/min) over a time course of 20 min. **[^18^F]E=SF** was captured on the short silica SPE cartridge (RCY 27 ± 6%, *n* = 2) and eluted with MeCN (1 mL), with an elution efficiency of 88–90% (Table 3).

For labelling using our recently described microlitre scale radiofluorination approach in HPLC vials (2 mL screw top vial, Agilent) [19], 50 µL aliquots of **[^18^F]E=SF** were taken, mixed with 50 µL of either EtdiTos/TEAB (10 mg/mL EtdiTos and 2 mg/mL TEAB in MeCN) or EtdiTos/KHCO_3_^.^K_222_ (8 mg/mL EtdiTos and 53 mM KHCO_3_^.^K_222_ in MeCN), and heated at 90 °C for 10 min, followed by deactivation and dilution with 200 µL MeCN/H_2_O and analysis of RCC by radio-TLC (Table 4).

#### 4.1.3. [^18^F]Ethanesulfonyl Fluoride ([^18^F]E-SF)

Manual radiosynthesis of [^18^F]ethanesulfonyl fluoride (**[^18^F]E-SF**) using [^18^F]TEAF: Elution of [^18^F]fluoride with TEAB solution, azeotropic drying and trapping on short and long silica SPE cartridges was performed as described above for **[^18^F]E=SF** using 2,4,6-trichlorophenyl ethanesulfonate (TCP-ethane, 20 mg/mL, 100 µL) in DMSO. **[^18^F]E-SF** was captured on the first (short, RCY 68 ± 12% (*n* = 2), Supplementary Information Table S1) but not the second (long) silica SPE cartridge. **[^18^F]E-SF** was eluted from the short cartridge with MeCN (1 mL), with an elution efficiency of 95%.

Optimised radiosynthesis of [^18^F]ethanesulfonyl fluoride (**[^18^F]E-SF**): Cyclotron-produced [^18^F]fluoride (400–700 MBq) was trapped on a waters QMA cartridge, washed with 1 mL MeCN and eluted with 1 mL HYFE solution. An aliquot of the eluted [^18^F]fluoride (100 µL) was dried down at 90 °C for 3–4 min, applying a stream of helium, and 2,4,6-trichlorophenyl ethanesulfonate (TCP-ethane, 100 µL, 20 mg/mL in DMSO) was added. The closed vial was connected by a needle to a Silica Plus light cartridge from the male side, which was followed by a transfer line to the final gas waste bag. The mixture was reacted at 100 °C, and the product was carried out of the reactor under N_2_ flow (medium flow) over a time course of 20 min. **[^18^F]E-SF** was captured on the Silica Plus light cartridge and eluted with MeCN (1 mL), providing the relay reagent at an RCY of 76 ± 23% (*n* = 6) (Table 5, Supplementary Information Table S1).

For labelling using the microlitre scale radiofluorination approach in HPLC vials (2 mL screw top vial, Agilent) [19], **[^18^F]E-SF** aliquots of 50 µL were taken and mixed with 50 µL of either EtdiTos/TEAB (10 mg/mL EtdiTos and 2 mg/mL TEAB in MeCN), EtdiTos/KHCO_3_^.^K_222_(8 mg/mL EtdiTos and 53 mM KHCO_3_^.^K_222_ in MeCN), or TMABTf/TEAB (10 mg/mL TMABTf and 2 mg/mL TEAB in MeCN) and heated at 90 °C for 10 min, followed by deactivation and dilution with 200 µL MeCN/H_2_O and analysis of RCC by radio-TLC or radio-HPLC (Table 6).

## 5. Conclusions

^18^F-relay reagents continue to offer promising tools for enabling modular, late-stage ^18^F-fluorination of delicate or high-value base-sensitive substrates, simplifying and decentralising fluorine-18 radiochemistry, with potential to expand access to PET tracers beyond traditional cyclotron-equipped centres. In this work, we demonstrated that **[^18^F]TfF**, **[^18^F]E=SF** and the newly developed **[^18^F]E-SF** can be produced reliably using straightforward, operationally simple methods. **[^18^F]E-SF** in particular shows strong potential as a next-generation relay reagent. It benefits from accessible commercial precursor supply and high process yields, retaining the possibility of SPE trapping and transport to satellite labs comparable to **[^18^F]E=SF**. This feature directly supports the development of simplified and decentralised strategies for the production of radiopharmaceuticals, as already common for single-photon computed tomography (SPECT) and PET imaging with generator-based radionuclides technetium-99m [24] and gallium-68 [25], respectively, thus addressing ongoing barriers to fluorine-18 access, especially in settings where infrastructure and radiochemical capacities are limited.

## Figures and Tables

**Table 1:** Radiochemical yields for the formation of **[^18^F]TfF**.

QMA Fluoride Elution Solution, Reaction Time	Activity Remaining in Generation Vial (d.c.)	Activity Trapped in Collection Vial (d.c.)
HYFE ¼, 10 min	0%	100%
HYFE ¼, 10 min	2%	96%
HYFE ¼, 10 min	1%	95%
HYFE, 10 min	4%	42%
HYFE, 6 min	1%	94%
HYFE, 5 min	13%	81%

**Table 2:** Radiochemical conversion for reactions towards [^18^F]fluorobenzaldehyde and [^18^F]FEtOTs using **[^18^F]TfF** determined by radio-HPLC or radio-TLC, respectively.

QMA Fluoride Elution Solution	Labelling Reaction	Radiochemical Conversion
HYFE ¼	EtdiTos	76 ± 1% (*n* = 3, TLC)
HYFE ¼	TMBATf	50 ± 4% (*n* = 3, HPLC)
HYFE	TMBATf	41 ± 4% (*n* = 3, HPLC)

**Table 3:** Radiochemical yields for the formation of **[^18^F]E=SF**.

QMA Fluoride Elution Solution	Activity Remaining in Generation Vial (d.c)	Trapped on Short Cartridge (d.c)	Trapped on Plus Cartridge (d.c)	Elution Efficiency
TEAB	60%	28%	5%	85%/83%
HYFE	60%	33%	Not used	88%
HYFE	Not determined	21%	Not used	90%

**Table 4:** Radiochemical conversion (RCC) for radiosynthesis of [^18^F]FEtOTs using **[^18^F]E=SF**.

QMA Fluoride Elution Solution	Labelling Reaction	Activator	Radiochemical Conversion
TEAB	EtdiTos	TEAB	45% (*n* = 1)
TEAB	EtdiTos	TEAB	68% (*n* = 1)
HYFE	EtdiTos	TEAB	42% (*n* = 1)
TEAB	EtdiTos	K_222_·KHCO_3_	73% (*n* = 1)
TEAB	EtdiTos	K_222_·KHCO_3_	77% (*n* = 1)
HYFE	EtdiTos	K_222_·KHCO_3_	73% (*n* = 1)

**Table 5:** Exemplary radiochemical yields for the formation of **[^18^F]E-SF** for the experiment where trapping on both short and plus Silica SPE cartridges was investigated.

QMA Fluoride Elution Solution	Activity Remaining in Generation Vial (d.c)	Trapped on Short Cartridge (d.c)	Trapped on Plus Cartridge (d.c)	Elution Efficiency
TEAB	17%	80%	2%	96%
HYFE	8%	98%	Not used	98%

**Table 6:** Radiochemical conversion for radiosynthesis of [^18^F]FEtOTs and 4-[^18^F]fluorobenzaldehyde using **[^18^F]E-SF**.

QMA Fluoride Elution Solution	Labelling Reaction	Activator	Radiochemical Conversion
TEAB	EtdiTos	TEAB	43% (*n* = 1)
HYFE	EtdiTos	TEAB	39% (*n* = 1)
TEAB	EtdiTos	K_222_·KHCO_3_	58% (*n* = 1)
HYFE	EtdiTos	K_222_·KHCO_3_	70% (*n* = 1)
TEAB	TMBATf	TEAB	60 ± 3% (*n* = 3)
HYFE	TMBATf	TEAB	72 ± 12% (*n* = 6)

## Data Availability

The original contributions presented in this study are included in the article/Supplementary Material. Further inquiries can be directed to the corresponding authors.

## Supplementary Materials

The following supporting information can be downloaded at https://www.mdpi.com/article/10.3390/ijms27093982/s1.

## Abbreviations

The following abbreviations are used in this manuscript:

[^18^F]E-SF	[^18^F]Ethanesulfonyl fluoride
[^18^F]E=SF	[^18^F]Ethenesulfonyl fluoride
[^18^F]TfF	[^18^F]Triflyl fluoride
d.c.	Decay corrected
EtdiTos	Ethylene ditosylate
HYFE	Solution of 29.4 mM K_2_CO_3_ and 58.9 mM K_222_ in MeCN/water (98/2)
HYFE ¼	Solution of 7.3 mM K_2_CO_3_ and 58.9 mM K_222_ in MeCN/water (97/3)
TMBATf	p-Trimethylammonium benzaldehyde triflate
