# Supplementary material for: Next-Gen 18F-Relay Reagent: Optimising [18F]Ethanesulfonyl Fluoride
Source: Int J Mol Sci. 2026 Apr 29;27(9):3982. doi: 10.3390/ijms27093982 (PMC13163567; doi:10.3390/ijms27093982)
Supplement: Supplementary file 1 [file ijms-27-03982-s001.zip › ijms-4263394-supplementary.pdf]

Supplementary Material

Next-Gen ^{18}F-Relay Reagent: Optimising [^{18}F]Ethanesulfonyl Fluoride

Margaret L. Aulsebrook [1*], Giancarlo Pascali [2], Kellie L. Tuck [3], Parisa T. Rashid [1,3], Manja Kubeil [4,5], Christoph E. Hagemeyer [1,6], Jens Pietzsch [4,7] and Markus Laube [4*]

Synthesis and purification of 2,4,6-trichlorophenyl ethenesulfonate:

Adapting the methods employed by Liu et al. (2023) [1] and Zeng et al. (2022) [2], 2,4,6-trichlorophenol (98.7 mg, 0.5 mmol) and potassium hydroxide (28.1 mg, 0.5 mmol) were dissolved in 5 mL of MeCN. Subsequently, ethene sulfonyl fluoride (66 mg, 0.6 mmol) was added to the round-bottom flask. The reaction mixture was stirred at 50 °C for 9 hours. Following this period, the mixture was extracted with ethyl acetate and an aqueous solution of sodium carbonate (10 mL each). The organic layer was then collected and concentrated under reduced pressure, yielding the target compound as a light green solid (69.6 mg, 48% yield). ^{1}H-NMR (400 MHz, $CDCl_3$) δ 7.40 (s, 2H), 6.94 (dd, J = 16.6, 9.9 Hz, 1H), 6.56 (dd, J = 16.6, 0.9 Hz, 1H), 6.26 (dd, J = 9.9, 1.0 Hz, 1H). ^{13}C-NMR ($CDCl_3$) δ: 129.16, 130.74, 131.31, 133.08, 133.88, 141.95.

Nuclear magnetic resonance (NMR) spectroscopy of 2,4,6-trichlorophenyl ethenesulfonate was performed on a Bruker Sample Xpress 400 (1H at 400.13 MHz). Spectra were processed using MestReNova 14.2.0 software. The NMR spectra was recorded at 25 °C. All chemical shifts (δ) are reported in parts per million (ppm) and referenced to residual solvent peaks relative to TMS. Coupling constants (J) are reported in Hz.

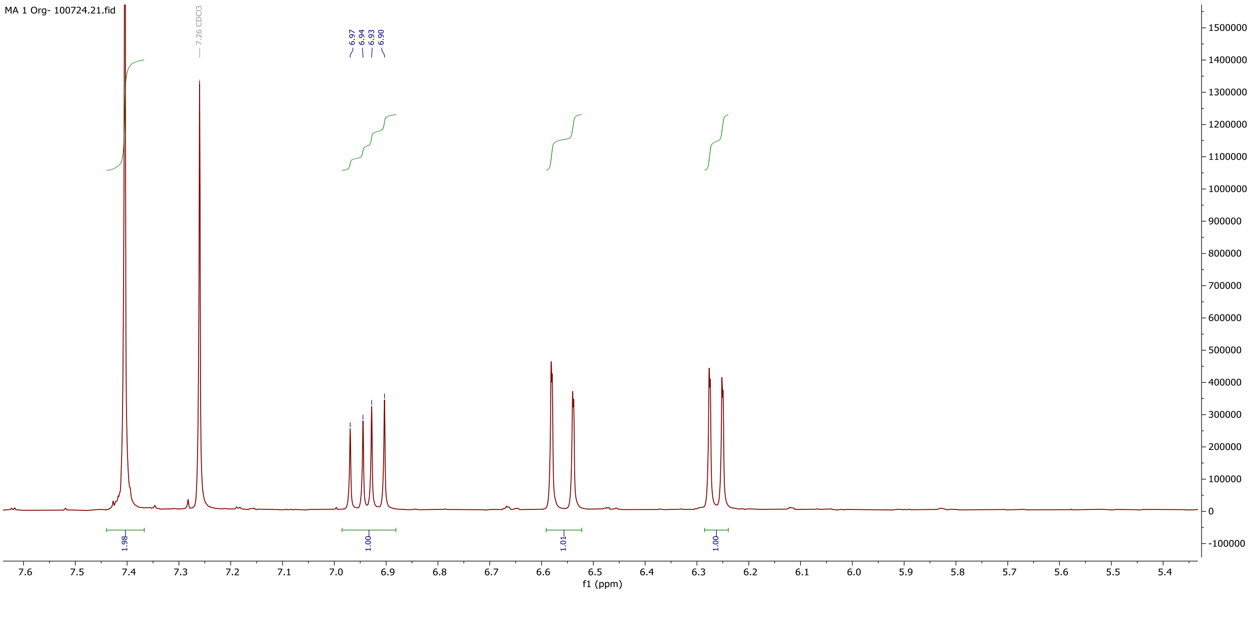

Figure S1: The ^{1}H NMR spectrum (400 MHz) of 2,4,6-trichlorophenyl ethenesulfonate, in deuterated chloroform.

Detailed results for RCY of [^{18}F]E-SF

Table S1. Radiochemical yields obtained for [^{18}F]E-SF using either TEAB or HYFE.

RCY [%]	#1	#2	#3	#4	#5	#6	Mean ± SD (n)
TEAB	56	80					68.0 ± 12.0 (n=2)
HYFE	87	81	28	96	89	73	75.7 ± 22.5 (n=6)

[1] Liu, M.; Tang, W.; Qin, H.L. Discovery of (*E*)-2-Methoxyethene-1-sulfonyl Fluoride for the Construction of Enaminyl Sulfonyl Fluoride. *J. Org. Chem.* **2023**, *88(3)*, 1909-1917.

[2] Zeng, Y.Z.; Wang, J.B.; Qin, H.L. A reductive dehalogenative process for chemo-and stereoselective synthesis of 1, 3-dienylsulfonyl fluorides. *Org. Biomol. Chem.* **2022**, *20(39)*, 7776-7780.
